# Photoacoustic Nanotagging for In Vivo Tracking and Optimization of Macrophage Immunotherapy in Solid Tumors

**DOI:** 10.1002/adma.74976

**Published:** 2026-09-13

**Authors:** Seoyoon Song, Melissa Cadena, Myeongsoo Kim, Euisuk Chung, Jeungyoon Lee, Zlata Brushevich, Seonghun Shin, Paul S. Pelkowski, Stanislav Y. Emelianov

**Affiliations:** ^1^ Wallace H. Coulter Department of Biomedical Engineering Georgia Institute of Technology and Emory University School of Medicine Atlanta Georgia USA; ^2^ Petit Institute for Bioengineering and Biosciences Georgia Institute of Technology Atlanta Georgia USA; ^3^ School of Electrical and Computer Engineering Georgia Institute of Technology Atlanta Georgia USA

**Keywords:** image‐guided therapy, macrophages, solid tumor immunotherapy, ultrasound/photoacoustic (US/PA) imaging

## Abstract

Macrophage‐based immunotherapy holds promise for solid tumors, yet critical limitations remain such as narrow insight into their in vivo localization and persistence of therapeutic macrophages for assessing their treatment efficacy. We report an immunostimulatory macrophage nanotagging strategy for image‐guided optimization of macrophage‐based solid tumor immunotherapy. Nanotags employ the branched gold nanostructure for efficient absorption in the near‐infrared range for photoacoustic contrast. Subsequent conjugation with interferon gamma (IFNγ) confers macrophage polarization capability into an antitumor phenotype that is superior to free IFNγ without sacrificing the viability. Following systemic administration, nanotagged macrophages are non‐invasively monitored using ultrasound‐guided photoacoustic (US/PA) imaging enabling localization of the infused macrophages and their persistence in the solid tumor mass in real‐time. Based on the observed limited persistence of systemically administered macrophages, lipopolysaccharide is introduced as a local supportive agent. The combination approach enhances macrophage retention and therapeutic efficacy. Rechallenged mice remain tumor‐free, indicating adaptive immune memory. Together, this approach offers a platform for longitudinal monitoring of therapeutic macrophages and imaging‐guided optimization of macrophage‐based solid tumor immunotherapy.

## Graphical abstract

Macrophage‐based immunotherapy for solid tumors is limited by poor tracking of cell localization and persistence. This study introduces nanotagged macrophages enabling near‐infrared photoacoustic imaging for real‐time monitoring. IFNγ‐conjugated tags enhance antitumor polarization without harming viability. Imaging‐informed therapy adjustments, including a combination approach, improve retention and efficacy, achieving complete remission and long‐term immune memory in mice.

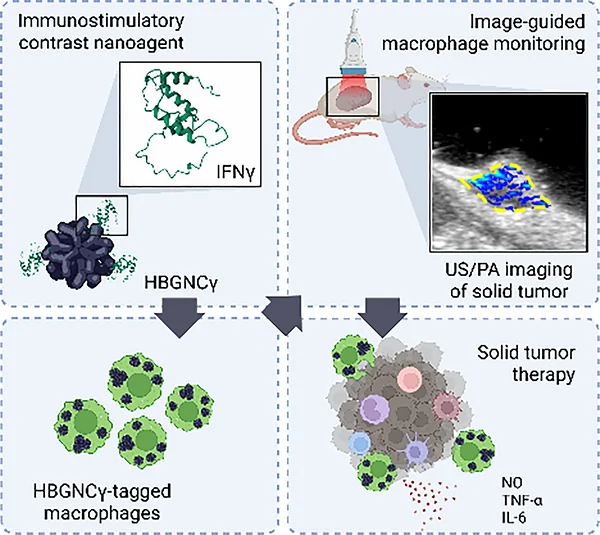

## 1 Introduction

Despite advancements in cellular immunotherapy, including the chimeric antigen receptor (CAR) T cells [1, 2], solid tumors remain unconquered even though they account for more than 90% of all cancer cases [3]. Current T cell‐weighed tumor therapies face two major challenges: (1) Limited localization/infiltration into solid tumors [4] and (2) relapse [5, 6, 7]. These challenges can potentially be addressed by employing macrophages as a cellular modality for solid tumor therapy, where macrophages are tissue‐resident cells that home to tumors and can engage adaptive immunity [8]—thereby addressing concerns of solid tumor localization and relapse.

For reinforced antitumor functions, macrophages have been engineered via different approaches including genetic engineering for the expression of CAR [9, 10, 11, 12] therapeutic proteins [13] and antitumor‐priming using cytokines/antibodies [14, 15, 16]. These approaches have shown promise in preclinical studies, with some in clinical trials [17]. However, a major unmet need in these emerging approaches is the ability to monitor in vivo trafficking and fate of engineered macrophages, because their localization and persistence are associated with subsequent therapeutic efficacy [13, 16, 18, 19]. Alternatively, therapeutic nanoparticle formulations with imaging capabilities, including aggregation‐induced emission luminogens, have been demonstrated. Fluorescence/photoacoustic image‐guidance, tumor microenvironment (TME) remodeling, and antimetastatic effects have been achieved, attributed to the spectral features and the use of macrophage membranes [20, 21, 22, 23]. However, since extracted membranes were used, active macrophages’ cellular functions, including antigen presentation and cytokine secretions, might have been limited.

Monitoring the trafficking of infused therapeutic cells via imaging techniques enables the assessment of their accumulation and persistence, which provides an estimate of therapeutic efficacy. Moreover, imaging signals can guide data‐driven adjustments to therapy, including modifying injection regimes or adding supportive agents for enhanced therapeutic outcomes [24, 25]. Ultrasound‐guided photoacoustic (US/PA) imaging is a suitable non‐invasive and real‐time imaging technique, where US imaging provides high‐resolution anatomical information [26] while PA imaging can detect signals from infused cells tagged with exogenous contrast agents [27, 28, 29]. Thus, US/PA monitoring of contrast agent‐tagged therapeutic cells can potentiate the therapy.

Here, we incorporate a PA contrast nanoparticle formulation into macrophages to exploit both image‐guidance and living macrophages’ antitumor functions via a single approach. An immunostimulatory PA contrast nanotag, hyperbranched gold nanoconstructs‐interferon gamma (IFNγ) conjugate (HBGNCγ), efficiently tag macrophages with ∼100% efficiency following simple incubation, which compares with genetic engineering of macrophages that has lower transfection efficiency and longer time to engineered macrophages. We demonstrate the HBGNCγ formulation's ability to program the phenotype of macrophages into antitumor phenotypes. Following systemic injection of HBGNCγ‐tagged macrophages into solid tumor‐bearing mice, macrophages’ trafficking into the tumor is monitored via US/PA imaging. Based on longitudinal imaging signals, a supportive agent, lipopolysaccharide (LPS), is added to the therapy regime. A therapeutic outcome‐indicative imaging response—enhanced persistence of macrophages in the tumor—is observed in combination therapy with LPS, leading to tumor inhibition effects. Mice with tumor regressions are protected against rechallenge, implying the involvement of adaptive immunity. Our strategy of tagging macrophages with immunostimulatory PA imaging probes provides insights into developing image‐guided, nanoparticle‐based, and macrophage‐driven solid tumor therapies.

## 2 Results

### 2.1 Immunostimulatory PA Nanotags

Given that near‐infrared (NIR) light penetrates deeply into biological tissues, contrast agents with high NIR absorption are desired for effective PA imaging [30, 31, 32]. We have synthesized hyperbranched gold nanoconstruct (HBGNC) as a PA contrast agent that exhibits high absorption profile in the NIR range [33]. In this study, macrophages are tagged with HBGNCs conjugated with IFNγ, namely HBGNCγ, for phenotypic modulation into antitumor phenotypes and image‐guided solid tumor immunotherapy following systemic injection (Figure 1a). The HBGNCs were prepared from 35 nm gold nanospheres via seed‐mediated anisotropic growth of branched structures (Figure 1b–d). The synthesized HBGNCs displayed high absorption in the NIR region, and the absorption spectra remained unchanged over a week, implying stable longitudinal imaging capability (Figure S1). The hydrodynamic diameter had negligible changes after conjugation with IFNγ (Figure 1e), while there was a slight decrease in zeta potential (Figure 1f). The conjugation of IFNγ did not alter the absorption properties of HBGNCs (Figure S2).

**FIGURE 1:**
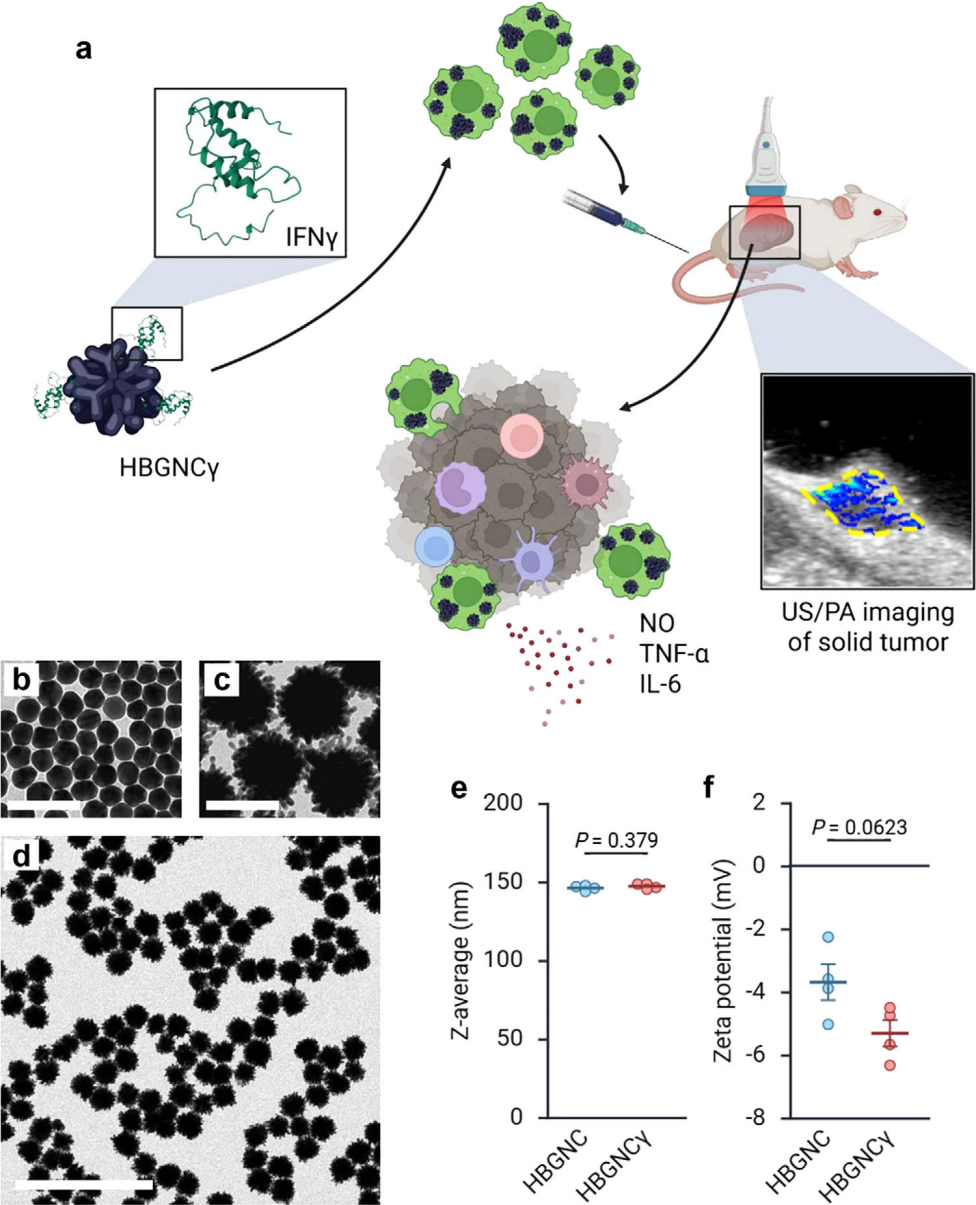
Study design and nanotag synthesis. (a) Schematic illustration of study design. HBGNCγ‐tagged macrophages were infused into tumor‐bearing mice for image‐guided solid tumor immunotherapy. (b–d) TEM images of 35 nm gold nanosphere seeds (b) and HBGNCs (c, d). (e) Hydrodynamic diameter of HBGNC and HBGNCγ. Data represent the mean with SEM (*n* = 4). Unpaired two‐tailed *t*‐test. (f) Zeta potential of HBGNC and HBGNCγ. Data represent the mean with SEM (*n* = 4). Unpaired two‐tailed *t*‐test. Scale bars for (b, c) are 100 and 500 nm for (d).

### 2.2 HBGNC Tagging and Antitumor Phenotypic Modulation

We first performed HBGNC tagging experiments to investigate how tagging affects macrophages. Murine bone marrow‐derived macrophages were incubated with HBGNCs of different concentrations (optical density, OD at 800 nm, as a measure). After overnight incubation, tagged macrophages were evaluated for viability. Until the OD 1 concentration, viability remained ∼100% but began to decrease from OD 2 (Figure 2a). The tagged cells remained fully viable over days when tagged with OD 1 concentration (Figure S3). Based on this observation, all further tagging conditions were fixed at OD 1 unless mentioned otherwise. Tagged particles were observed from OD 0.25 under optical microscopy images and were most prominent in the OD 1 condition, with increased sizes of the tagged cells (Figure 2b). Representative flow cytometry plots of the main macrophage population at OD 0 and 1 show increases in both forward scatter (FSC) and side scatter (SSC) values, indicating increases in both cell size and granularity (Figure 2c). Depending on the concentration of the HBGNCs, both FSC and SSC values linearly increased (Figure 2d,e).

**FIGURE 2:**
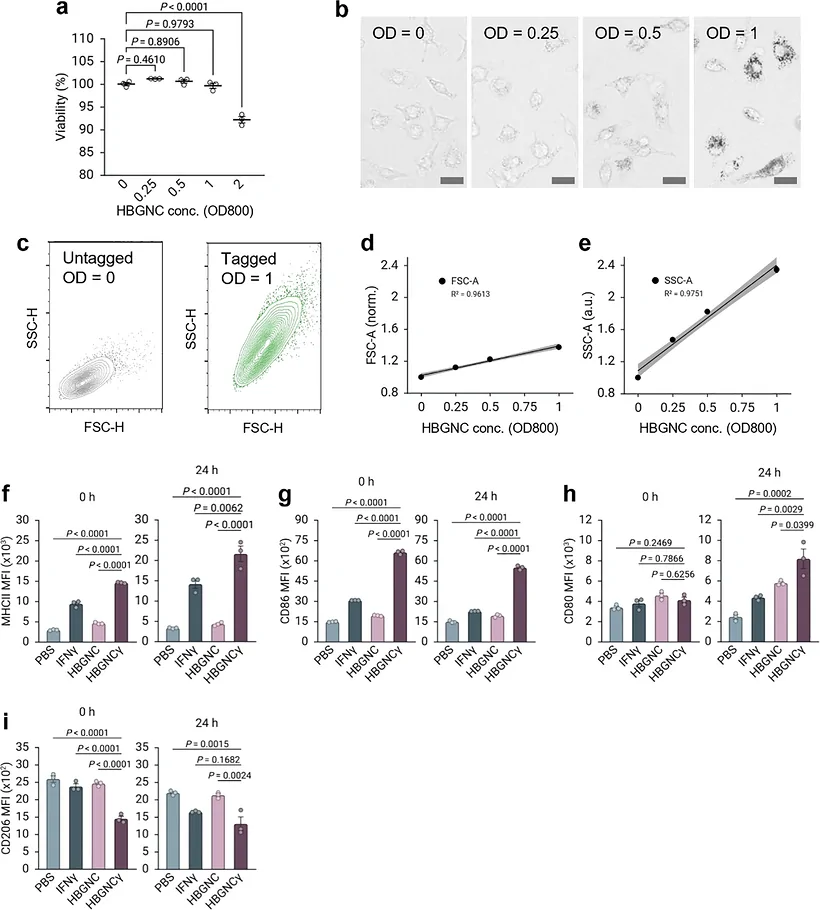
Morphological changes and immunological activation of macrophages tagged with HBGNC and HBGNCγ. (a) Viability of macrophages tagged with different concentrations of HBGNCs (overnight incubation). Data represent the mean with SEM (*n* = 3). One‐way ANOVA with Tukey's multiple comparisons test. (b) Representative optical microscopy images of macrophages tagged with different concentrations of HBGNCs. Scale bar: 20 µm. (c) Flow cytometry plot (SSC vs. FSC) of macrophages with (OD 1) and without (OD 0) HBGNC tagging. (d, e) Scatter plot of tagged macrophages size (FSC) (d) and granularity (SSC, normalized to 0 HBGNC concentration) (e). Data represent the mean with SEM (*n* = 3). Shaded area represents the 95% confidence interval. (f–i) Expression levels of macrophage antitumor markers MHCII, CD86, and CD80 (f–h) and pro‐tumor marker CD206 (i). Data represent the mean with SEM (*n* = 3). One‐way ANOVA with Tukey's multiple comparisons test.

We next analyzed the expression levels of antitumor (MHCII, CD86, and CD80) and pro‐tumor (CD206) markers of macrophages upon different treatments of control (PBS), free IFNγ, HBGNC, and HBGNCγ. IFNγ concentration was 100 ng/mL for both IFNγ and HBGNCγ groups. HBGNC‐treated macrophages showed similar expression levels of MHCII and CD86 to the PBS group at both 0 and 24 h points after each treatment (Figure 2f,g). Meanwhile, HBGNCγ group macrophages showed the highest expression levels of all three antitumor markers (Figure 2f–h). HBGNCγ group macrophages had the lowest expression level of CD206 as well (Figure 2i). HBGNCγ outperformed free IFNγ in most concentrations (0, 10, 50, and 100 ng/mL) (Figure S4a–d). These results collectively indicate the phenotypic modulatory performance of the HBGNCγ formulation. Further, macrophages treated or tagged with PBS, IFNγ, HBGNC, or HBGNCγ are referred to as PBS‐Mac, IFNγ‐Mac, HBGNC‐Mac, and HBGNCγ‐Mac, respectively.

### 2.3 Superior Functional Responses of Tagged Macrophages

It is important to ensure that functional responses of the engineered cells do not significantly diminish. Therefore, we tested macrophages’ functional responses after tagging with HBGNCs, including the responses towards migratory and inflammatory signals. For the functional responses towards migratory cues, a transwell migration assay was performed where fluorescently stained macrophages were placed in the apical chamber, and a migration‐promoting molecule, monocyte chemoattractant protein‐1 (MCP‐1), was placed in the media on the basolateral side (Figure 3a). After 6 or 24 h, remaining macrophages in the apical side were removed, and the basolateral side of the transwell was imaged. While the migration of HBGNC‐Mac and HBGNCγ‐Mac did not significantly increase compared to the PBS‐Mac group at the 6 h point (Figure S5a,b), the number of migrated macrophages was fourfold greater for both HBGNC‐Mac and HBGNCγ‐Mac at the 24 h point (Figure 3b,c). Results suggest that HBGNC tagging not only did not negatively affect migratory behavior due to increased cell size but also had positive effects.

**FIGURE 3:**
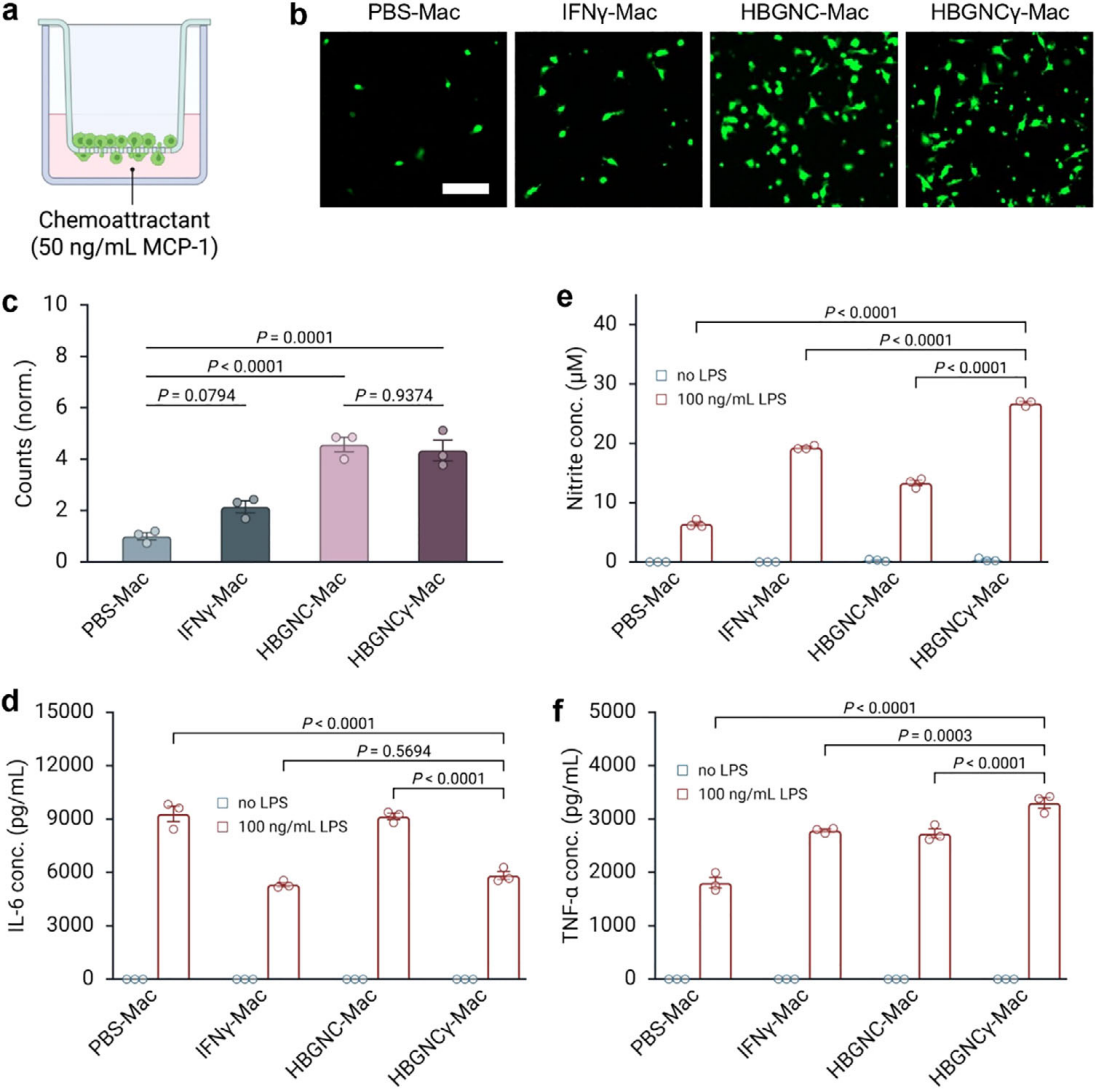
Responses of tagged macrophages toward migratory and inflammatory cues. (a) Schematic illustration of the migration experiment. Macrophages were placed in the apical chamber. Migrated macrophages (the basolateral side) were imaged. (b) Representative confocal microscopy images of the migrated macrophages at the 24‐h point. Scale bar: 100 µm. (c) Number of migrated macrophages (normalized to PBS‐Mac group) across different treatment groups at 24‐h post‐incubation. Data represent the mean with SEM (*n* = 3; each data point represents the average number of macrophages from three different random points). One‐way ANOVA with Tukey's multiple comparisons test. (d–f) Inflammatory/antitumor responses of macrophages across different treatment groups toward 0 and 100 ng/mL LPS for IL‐6 (d), nitrite (e), and TNF‐α (f) at 24 h post‐LPS treatment. Data represent the mean with SEM (*n* = 3). Two‐way ANOVA with Tukey's multiple comparisons test.

IFNγ‐primed macrophages are known to become hyperresponsive to immunological stimuli, releasing greater amounts of inflammatory molecules/cytokines that are associated with antitumor responses [34]. To investigate this, we used LPS, a bacteria‐derived macrophage stimulant that is often used in combination in immunotherapy [34, 35, 36]. Macrophages were incubated with and without 100 ng/mL LPS to PBS‐Mac, IFNγ‐Mac, HBGNC‐Mac, or HBGNCγ‐Mac treatment and measured the concentration of representative inflammation mediators secreted by macrophages—nitrite, IL‐6, and TNF‐α. No measurable amount was detected for all three species in the group without LPS. For IL‐6, both PBS‐Mac and HBGNC‐Mac groups showed similar expression levels that were greater than IFNγ‐treated groups of IFNγ‐Mac and HBGNCγ‐Mac (Figure 3d and Figure S6a). For nitrite and TNF‐α, the HBGNCγ‐Mac group secreted the highest concentration among all groups (Figure 3e,f and Figure S6b,c). Moreover, responses of macrophages in all groups against monophosphoryl lipid A (MPLA), a detoxified derivative of lipid A from LPS with approved use in clinic [37], showed comparable levels of nitrite, IL‐6, and TNF‐α concentrations (Figure S7a–c). These results suggest a potentially beneficial synergism of HBGNCγ‐Mac with adjuvants in a combination therapy regime.

### 2.4 Spectral Features of Tagged Macrophages

Based on experimental observations of antitumor polarization and superior functional responses following tagging, we validated that HBGNC‐Mac can be visualized using a US/PA imaging system. Tagged macrophages were embedded in a tissue‐mimicking phantom [38] for US/PA imaging (Figure 4a). First, macrophages were tagged with different concentrations of HBGNCs (OD 0 to 1) and imaged at a final cell concentration of 2,000 cells/µL. In US/PA overlay images, macrophages were visible with a strong PA signal from tagging concentration of OD 0.5 (Figure 4b). PA signal was highly linear with tagging concentration (Figure 4c). Subsequently, tagging concentration was fixed at OD 1 and US/PA imaging was performed with different cell concentrations. PA spectral scan in the 700–900 nm spectral range showed cell concentration‐dependent PA signal amplitude (Figure 4d). US/PA overlay images show that tagged macrophages can be detected from 250 cells/µL concentration at an 800 nm imaging wavelength (Figure 4e). PA signal versus cell concentration graph displays linearity between the two (Figure 4f). Next, photostability of the HBGNCs in the tagged HBGNC‐Mac state was investigated to validate PA signal stability. PA signals were maintained without signal decay for 4000 laser pulses, which correspond to 1000 image frames (Figure 4g). Moreover, the PA signal was well maintained in the tagged status over days (Figure S8a,b), and the viability of the HBGNC‐Mac did not decrease after laser irradiation (Figure S9). In line with these observations, extinction of the HBGNC‐Mac showed concentration‐dependency, and the extinction and cell concentration had a linear relationship (Figure 4h,i). These results indicate that macrophages can be tagged with HBGNCs to generate a robust PA signal for longitudinal imaging.

**FIGURE 4:**
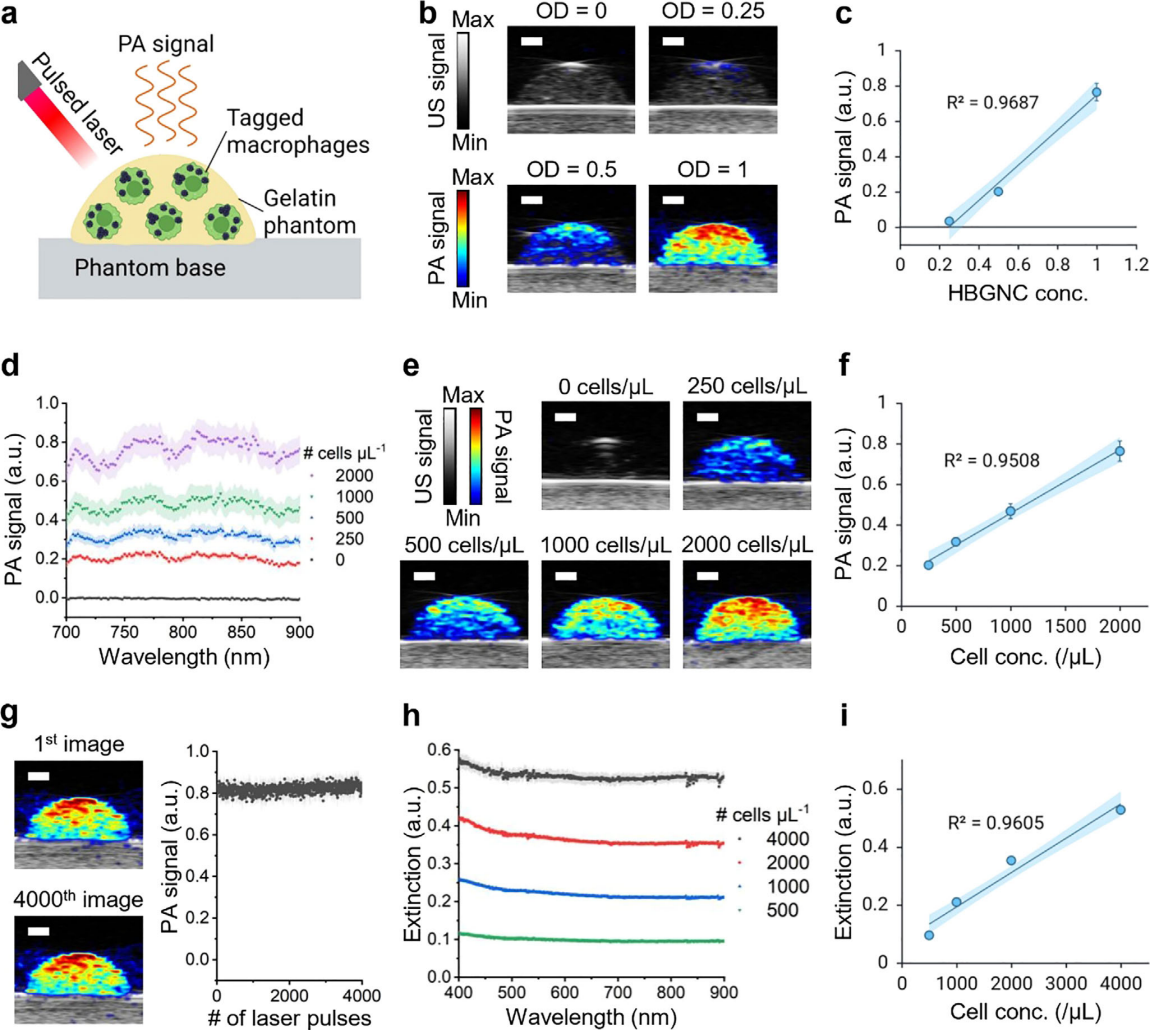
Optical and acoustic responses of tagged macrophages. (a) Schematic illustration of the PA imaging experiment. HBGNC‐tagged macrophages were embedded in gelatin dome phantoms and imaged. (b) US/PA overlay images of tagged macrophages with different HBGNC concentrations at a fixed cell concentration (2000 cells/µL). (c) PA signal versus HBGNC concentration plot from images in (b). Data represent the mean with SEM (*n* = 3). (d) PA spectra of HBGNC‐tagged macrophages (OD 1 concentration) with different cell concentrations over the NIR I range. Shaded area around the lines represents SD (*n* = 3). (e) US/PA overlay images of tagged macrophages with different cell concentrations at fixed HBGNC concentration (OD 1). (f) PA signal versus cell concentration plot from images in (e). Data represent the mean with SEM (*n* = 3). (g) Photostability of HBGNCs tagged onto macrophages over 4000 laser pulses. Images after the first and 4000th pulses are shown (left) with PA signal quantification (right). Shaded area around the data points represents SD (*n* = 3). (h) Extinction of HBGNC‐tagged macrophages (OD 1) with different cell concentrations. Shaded area around the data points represents SD (*n* = 3). (i) Extinction versus cell concentration plot from (h). Data represent the mean with SEM (*n* = 3). For (c, f, and i), the shaded area around the fitted line indicates the 95% confidence interval. Scale bar for dome images: 1 mm.

### 2.5 US/PA Visualization of Injected Macrophages in Solid Tumor

Building on our in vitro findings, we proceeded to an in vivo study to verify our strategy in a more physiologically complex environment. A tumor‐bearing mouse model was used to evaluate the imaging and therapeutic potential of the tagged macrophages. A subcutaneous CT26 tumor was established in the right flank on Day 0, and US/PA imaging was performed every other day from Day 5 for a total of five times. Macrophages were intratumorally injected on Day 6 (Figure S10a). While PBS‐Mac and IFNγ‐Mac groups did not show any appreciable PA signal over the course of imaging sessions, HBGNC‐Mac and HBGNCγ‐Mac groups displayed strong PA signal (Figure S10b). PA signal quantification showed almost identical levels of PA signal on Day 7 and slowly decreased over time (Figure S10c). For tumor volumes, the HBGNCγ‐Mac group showed the slowest growth (Figure S10d). Local injections in the tumor did not lead to any changes in the animals’ body weights (Figure S10e).

Then, a systemic administration study was performed. Macrophages were systemically injected via the tail vein on Day 6, and US/PA imaging was performed four times, similarly to the local injection study (Figure 5a). Appreciable PA signal was only observed in US/PA images of the HBGNCγ‐Mac group on day 7 (Figure 5b,c). However, negligible effects on tumor volumes were observed (Figure 5d), yet systemic injections did not cause any changes in mice body weights (Figure S11).

**FIGURE 5:**
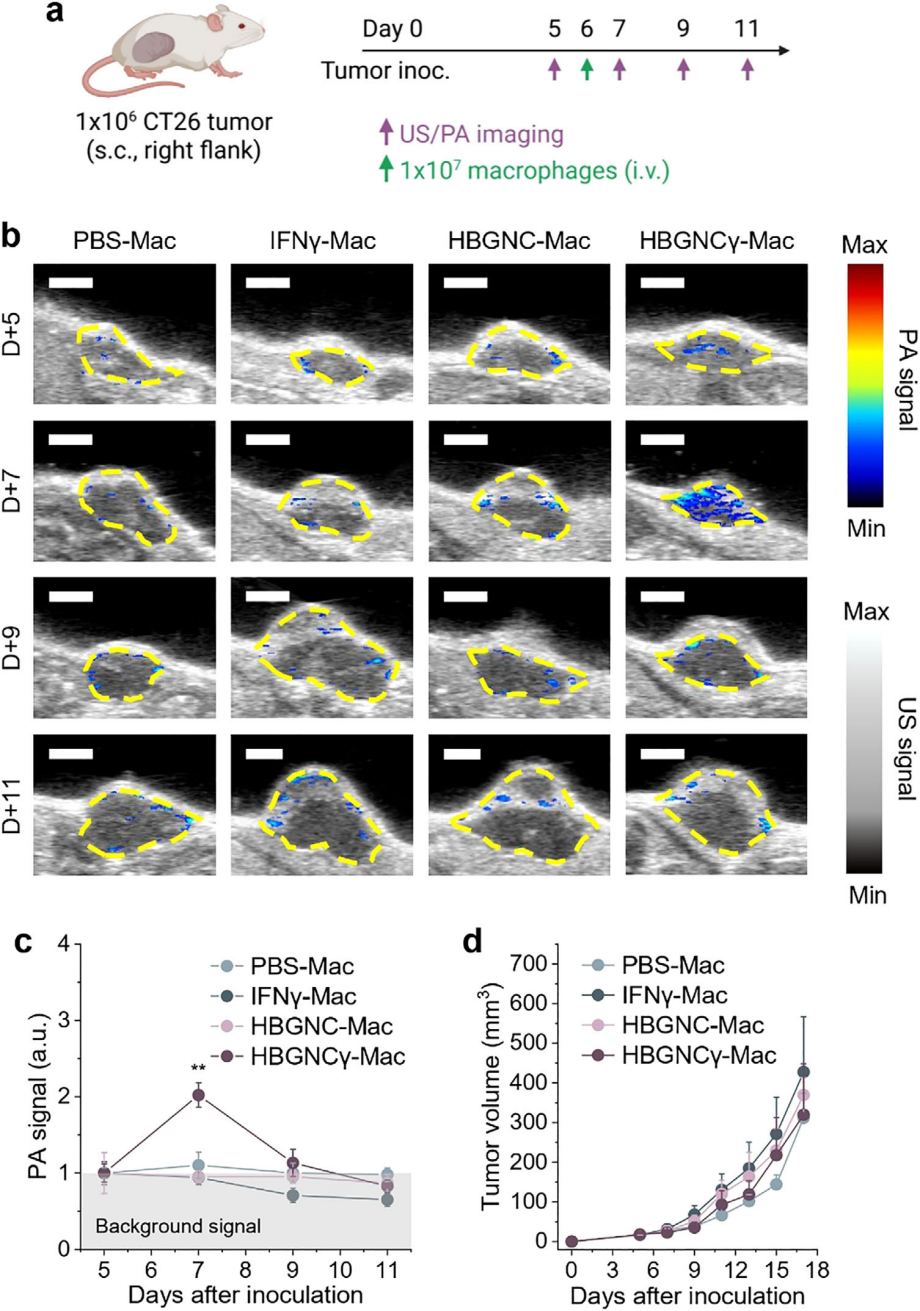
US/PA monitoring of solid tumor localization and persistence of tagged macrophages. (a) Treatment and imaging regime. Subcutaneous CT26 tumor was inoculated on Day 0. US/PA imaging was performed every other day from Day 5 post‐inoculation for a total of four times. Macrophages were intravenously injected on Day 6. (b) Representative US/PA overlay images of the tumors with different treatments. Dotted lines indicate the tumor periphery. Scale bar: 2 mm. (c) Quantified PA signals from (b). Data represent the mean with SEM. Two‐way repeated measure ANOVA with Bonferroni's pairwise comparisons on Day 7 between PBS‐Mac and HBGNCγ‐Mac. **p < 0.01. (d) Tumor volume monitoring. Data represent the mean with SEM. *n* = 4 mice per group for (b–d).

### 2.6 US/PA‐Informed Combination Immunotherapy

Therapeutic strategies should be refined upon suboptimal efficacy. From the previous experiment, we were informed by US/PA imaging that systemic injection of HBGNCγ‐Mac alone is ineffective (Figure 5d). US/PA images showed limited localization and persistence of macrophages in the tumor (Figure 5b,c). Thus, we sought to adjust the therapy regime using a combination therapy approach. To recruit more macrophages and/or make the recruited macrophages persist longer in the tumor, local injections of LPS were added to the same tumor model and therapy regime with systemic administration alone (Figure 6a). Local injections of LPS as outlined in Figure 6a did not have tumor inhibition effects (Figure S12). In this adjusted combination therapy regime, both HBGNC‐Mac and HBGNCγ‐Mac groups displayed enhanced persistence of macrophages in the tumor (Figure 6b,c, and Figure S13), yet noticeable tumor inhibition effects were observed only in the HBGNCγ‐Mac group (Figure 6d). Accordingly, the survival rate of the mice in the HBGNCγ‐Mac group was the highest (Figure 6e). The combination therapy did not have significant effects on animals’ body weights as well as body conditioning scores [39] (Figure S14a,b). The surviving mice were rechallenged with the same CT26 cells, and none of the surviving mice showed tumor growth (Figure 6f). The rechallenged mice were further evaluated for anti‐metastatic effect, as it evidences the induction of systemic immune memory. Along with fresh control (no previous therapeutic treatment), the surviving mice from the HBGNCγ‐Mac group were intravenously injected with CT26 cells, mimicking a lung metastasis condition. Representative photographs and the number of metastatic nodules of the excised lungs after sacrifice show that while the lung from the fresh control group had numerous metastases, the lung from the HBGNCγ‐Mac did not show metastatic nodules (Figure S15a,b). Representative H&E staining images support the formation of metastatic nodules in the fresh control lungs, while normal lung morphology was observed in the HBGNCγ‐Mac group (Figure S15c). The survival and rechallenge experiments imply strong promise for combination therapy of HBGNCγ‐Mac macrophages with LPS. Moreover, the divergence in tumor volumes began from approximately Day 15, while PA signal in the tumor region was clearly identifiable from Day 7, potentially providing valuable information from image guidance early on to make appropriate changes to the regime.

**FIGURE 6:**
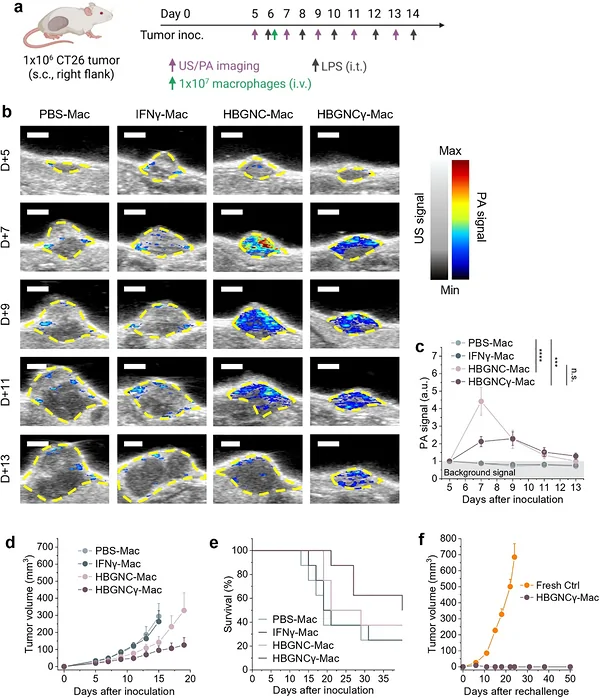
US/PA‐informed combination approach for solid tumor immunotherapy. (a) Treatment and imaging regime. Intratumoral injections of LPS were added. (b) Representative US/PA overlay images of the tumors with different treatments. Dotted lines indicate the tumor periphery. Scale bar: 2 mm. (c) Quantified PA signals from (b). Data represent the mean with SEM. Two‐way repeated measure ANOVA with Bonferroni's pairwise comparison. ***p < 0.001, ****p < 0.0001. (d) Tumor volume monitoring. Data represent the mean with SEM. (e) Survival rate. *n* = 8 mice per group from two independent experiments for (b–e). (f) Rechallenge experiment. Mice with tumor remissions from (d) were rechallenged with CT26 cells (*n* = 3 mice for Fresh Ctrl and *n* = 2 mice for HBGNCγ‐Mac). Data represent the mean with SEM.

Flow‐based immunological analysis of the tumor on Day 7 was performed to identify potential attributes for enhanced therapeutic effects in the HBGNCγ‐Mac group. The number of infused macrophages in the tumor showed a similar trend with PA signal of the tumor (Figure S16a). Although there were no clear differences across different cell populations and their activation status (Figure S16b–j), as LPS strongly skews immune cell activation [34, 35, 40], a significantly increased fraction of CD4^+^ T cells was observed (Figure S16k), suggesting a therapeutically beneficial innate‐adaptive immune coordination [41, 42].

## 3 Discussion

Myeloid lineage cells such as dendritic cells, monocytes, and macrophages pose several advantages over T cell‐based therapies in cancer immunotherapy, although myeloid cells have lower direct target‐specific cytotoxicity. (1) Myeloid cells can clear cancer cells by phagocytosis independent of antigen specificity, (2) process and present a number of neoantigen targets to direct adaptive immunity, and (3) recruit immune cells to sites and activate them through the secretion of various chemo/cytokines [8, 43, 44]. These features, especially the coordination of adaptive immunity, make it potentially overcome one of the most important challenges of cancer therapy, relapse. A number of studies have shown that myeloid cell‐based cancer immunotherapy can achieve resistance to tumor cell rechallenge after complete remissions [45, 46, 47, 48, 49, 50]. In line with these premises, we present a myeloid cell type, macrophage‐driven solid tumor immunotherapy strategy. Macrophages were tagged with an immunostimulatory PA nanotag for both antitumor effects and image‐guidance. Tagging was performed ex vivo, and macrophages were adoptively transferred to a solid tumor model. Local injections of LPS significantly enhanced the accumulation and retention of macrophages in the tumor tissue, providing a potential method to redirect macrophages into desired target tissues for potentiating the therapeutic efficacy. Moreover, mice with tumor remissions were protected against tumor cell rechallenge, indicating the involvement of adaptive immunity.

Macrophages generally do not expand once isolated/differentiated and cultured ex vivo. Because of this, genetic engineering/synthetic biology approaches can be more challenging [19] compared to T cells, as T cells can be expanded in culture. Meanwhile, due to their highly phagocytic nature, cellular nanoengineering—modulation of cellular functions using nanoparticles [51, 52]—is a suitable strategy to engineer macrophages. Efficient tagging of macrophages with nanoparticles can be achieved by simple incubation with nanoparticle‐containing media. Moreover, sizes and shapes of nanoparticles can be tuned to induce different polarization of macrophages [53], and different base materials can be selected for advanced applications, such as image‐guidance [26, 31]. A primary advantage of image‐guided therapy is its ability to provide real‐time insights into the spatiotemporal distribution and retention of therapeutic agents—thereby facilitating an accurate assessment of therapeutic delivery and the fate of therapy [24, 25, 27, 28]. Based on these insights, appropriate adjustments can be made for enhanced therapeutic efficacy, as in this study, where HBGNCγ treatment was supplemented with LPS. Therefore, tagging/engineering with nanoparticles can be a potent strategy in utilizing macrophages.

Although early clinical results showed limited therapeutic efficacy of adoptively transferred ex vivo cytokine‐primed antitumoral macrophages, this adoptive transfer strategy demonstrated the feasibility that it was well‐tolerated in humans [54, 55]. More recently, macrophages engineered with advanced technologies, including CARs and priming, are actively tested both preclinically and clinically [9, 10, 11, 12, 13, 14, 15, 16, 17]. Meanwhile, challenges hindering the clinical translation of nanoparticle/contrast agent‐tagging strategies are that often times they require complex design/synthesis and/or are inorganic material‐based [26, 31]. Although our current study employs an inorganic material (gold)‐based formulation, the same strategy of macrophage tagging, phenotype modulation, and image‐guidance can be achieved using organic and/or clinically proven materials for direct clinical relevance of the strategy. For instance, a perfluorocarbon‐based lipid shell formulation that can be imaged using deep‐penetrating ultrasound only, which our group is currently developing [56, 57, 58], can enhance clinical translatability, because not only does it use clinically proven materials but also allows imaging of deeper tissues compared to light‐based imaging technologies [59].

Overall, while some limitations remain, such as direct clinical translatability, our findings have the potential to provide insights into advancing current macrophage‐based solid tumor immunotherapies as well as the development of image‐guided cellular therapies.

## 4 Experimental Section

Confocal microscope LSM 900 (Carl Zeiss), installed at the Optical Microscopy Core at Georgia Tech, was used for all bright‐field and fluorescent images. Flow cytometers Cytoflex S (Beckman Coulter) and Aurora (Cytek), installed at the Cellular Analysis and Cytometry Core at Georgia Tech, were used for cytometry analysis. Hitachi HT7700 TEM is installed at the IMS Materials Characterization Facility at Georgia Tech. H&E staining was conducted at the histology core at Georgia Tech.

### 4.1 Reagents

Gold(III) chloride trihydrate (#520918), sodium citrate tribasic dihydrate (#C3434), sodium borohydride (#452882), potassium iodide (#221945), polyvinylpyrrolidone (#856568), L‐ascorbic acid (#A92902), silver nitrate (#209139), 2‐imminothiolane (#I6256), lipopolysaccharides from *Escherichia coli* O55:B5 (#L2880), and silica gel (#288500) were purchased from Sigma‐Aldrich. Gelatin Type A (#901771) was purchased from MP Biomedicals.

### 4.2 Cell Culture

Macrophages were prepared from mouse bone marrow cells. Bone marrow cells were isolated from 5–6 weeks old BALB/c mice as previously reported [60]. Briefly, bone marrow was collected from the tibia and femur via centrifugation at 10 000 rcf for 15 s, and erythrocytes were lysed with RBC lysis buffer (#420301, BioLegend). Bone marrow cells were differentiated in DMEM (#SH30243.01, Cytiva) supplemented with 1% P/S (#P4333, Sigma‐Aldrich), 10% FBS (#F0926, Sigma‐Aldrich), and 20 ng/mL M‐CSF (#576404, BioLegend) at 10^6^ cells/mL concentration for 6 days. On day 3, the same volume of fresh culture media was added. CT26 cells were cultured in RPMI 1640 medium (#SH30027, Cytiva) supplemented with 1% P/S and 10% FBS. All cells were maintained in a cell culture incubator at 37°C, 5% CO_2_.

### 4.3 HBGNC𝛾 Synthesis and Characterization

HBGNCs were synthesized using a three‐step method (3 nm gold seed → 35 nm gold nanospheres (GNS) → HBGNCs) as previously described [33]. To synthesize 3 nm gold seed, 0.5 mL of 5 mM gold(III) chloride trihydrate and 0.5 mL of 5 mM sodium citrate tribasic dihydrate were added to 9 mL of deionized water and stirred for 10 min. Subsequently, 0.3 mL of 100 mM sodium borohydride was added while stirring. This results in the synthesis of 3 nm gold seeds. To synthesize 35 nm spheres, 6 mL of 25 mM gold (III) chloride trihydrate, 2.4 mL of 200 mM potassium iodide, 6 mL of 5% (wt.) polyvinylpyrrolidone, and 2.4 mL of 100 mM L‐ascorbic acid were added to 48 mL of deionized water and stirred for 10 min. Then, 1.5 mL of the 3 nm gold seed prepared in the previous step was added while stirring and left for 30 min. The resulting GNS was washed three times with deionized water via centrifugation, and the concentration was adjusted to 3.32 nM. To synthesize HBGNCs, 2 mL of 3.32 nM GNS, 1 mL of 10% (wt.) polyvinylpyrrolidone, and 2.5 mL of 5 mM silver nitrate were added to 40 mL of deionized water and stirred for 5 min. Subsequently, 1 mL of 20 mM potassium iodide, 2.5 mL of 25 mM gold (III) chloride trihydrate, and 5 mL of 200 mM L‐ascorbic acid were added. The resulting HBGNCs were washed three times with deionized water via centrifugation. For conjugation, IFN𝛾 (#714006, BioLegend) was treated with 2‐imminothiolane for 30 min at RT. Then, thiol‐introduced IFN𝛾 was added to HBGNCs and left for 2 h at RT for the gold‐thiol reaction. For TEM imaging, 10 µL of 1/10 diluted product was added onto a copper TEM grid, dried, and imaged using Hitachi HT7700 TEM. Hydrodynamic diameter and zeta potential were measured in PBS using Zetasizer Nano‐ZS (Malvern).

### 4.4 Macrophage Tagging

*Macrophage morphology after tagging*: 5 × 10^5^ macrophages were plated in imaging glass‐bottom dishes (35 mm diameter) in 1 mL and left overnight. Macrophages were then treated with HBGNCs at OD 0, 0.25, 0.5, and 1 concentrations and incubated for another 16 h. Cells were then washed with PBS to remove any free particles and fixed with 4% paraformaldehyde for 20 min at RT. Then bright‐field images were obtained on an LSM 900 confocal microscope.

*Macrophages’ response to IFN𝛾and HBGNC𝛾*: 2 × 10^5^ macrophages were plated in 6‐well plates and left overnight. Either IFN𝛾 or HBGNC𝛾 with varying IFN𝛾 concentrations (0, 10, 50, and 100 ng/mL) were treated and incubated for 16 h. HBGNC concentration was fixed at OD_800_ = 1. After incubation, cells were washed and collected via scraping. Collected cells were stained with viability dye and surface markers (MHCII, CD80, CD86, and CD206) and analyzed by flow cytometry.

*Transwell migration assay*: 5 × 10^5^ macrophages were plated in 6‐well plates and incubated overnight. The cells were treated with either PBS, IFN𝛾 (100 ng/mL), HBGNC (OD 1), or HBGNC𝛾 for 16 h. After washing, cells were stained with 5 µM CellTracker Green CMFDA (#C2925, Invitrogen) in serum‐free media for 20 min. Excess dye was washed, and the cells were collected. Cells were counted and resuspended at 1 × 10^6^ cells/mL concentration. 100 µL of the suspension was transferred to the apical side of a transwell (#3422, Corning). 800 µL of 50 ng/mL MCP‐1 (#578402, BioLegend)‐containing media was added to the basolateral side. After 6 or 24 h, the transwell insert was washed with PBS, fixed in 4% PFA for 20 min at RT, and non‐migrated cells were removed with a cotton swab. The migrated cells were imaged using an LSM 900 confocal microscope.

*Responses toward LPS treatment*: 1 × 10^5^ macrophages were seeded in a 24‐well plate in 0.5 mL media and left overnight. Cells were treated with PBS, IFN𝛾, HBGNC, or HBGNC𝛾 and incubated for 16 h. IFN𝛾 concentration was 100 ng/mL, and HBGNC concentration was OD 1. Then, cells were washed, and the media was exchanged. Relevant wells received additional LPS treatment (100 ng/mL). After 6 or 24 h, the supernatant was collected and subjected to Griess assay (#G7921, Invitrogen), IL‐6 ELISA (#88‐7064‐88, Invitrogen), and TNF‐α ELISA (#88‐7324‐88, Invitrogen) following the manufacturer's protocol.

### 4.5 Animals

Animal experiments were reviewed and approved by Georgia Tech's Institutional Animal Care and Use Committee (IACUC, protocol #EMELIANOV‐A100281). Animals were housed in facilities managed by the Department of Animal Resources (DAR) at Georgia Tech. Animals were purchased from the Jackson Laboratory. Animals were regularly monitored and were euthanized when more than 20% of the body weight was lost, the longest dimension of the tumor reached 15 mm, or an ulcer was observed. The tumor volumes were measured using calipers. Tumor volumes were calculated as {(length) × (width)^2^ × 0.5}.

### 4.6 Tumor Model and Treatment

1 × 10^6^ CT26 cells in 100 µL of PBS were inoculated into the right flank of 6–8 weeks old female BALB/c mice. On day 6, 2 × 10^6^ macrophages were intratumorally injected or 1 × 10^7^ macrophages were injected via the tail vein. For combination therapy, LPS was intratumorally injected (2 µg per mouse per injection) every other day for a total of five times from day 6. For rechallenge study, 5 × 10^5^ CT26 cells were inoculated subcutaneously. For metastasis experiments, 1 × 10^6^ CT26 cells were intravenously injected via the tail vein. Two weeks later, mice were sacrificed and the lungs were isolated. The lungs were embedded in OCT compound and kept frozen. The frozen samples were sectioned and H&E‐stained.

### 4.7 Ultrasound‐Guided Photoacoustic Imaging

For in vitro phantom imaging, 5 × 10^5^ macrophages were plated in 6‐well plates and left overnight. HBGNC was treated with cells at varying concentrations (OD 0, 0.25, 0.5, and 1). After 16 h of incubation, cells were washed with PBS and collected via scraping. After resuspension, the solution was mixed with 16% (w/v) gelatin at a 1:1 ratio. Then, 20 µL of the mixture was transferred onto a gelatin/silica (8%, w/v) phantom base and incubated at 4°C for 30 min for gelation. The phantom was submerged in water and imaged. For in vivo imaging, tumor‐bearing mice were imaged under isoflurane‐induced anesthesia. US/PA imaging was performed using the Vevo2100/LAZR imaging system with LZ‐250 transducer (Fujifilm). The US B‐mode and PA gains were 18 and 40 dB, respectively. The imaging frequency for ultrasound was 21 MHz.

### 4.8 Flow Cytometry and Antibodies

Single‐cell suspensions of cell samples were prepared in cell staining buffer (#420201, BioLegend). The viability staining was performed with Zombie NIR fixable dye (#423105, BioLegend) for 15 min at room temperature. The cell suspension was treated with anti‐mouse CD16/32 antibody (1:50 dilution) (clone 93, BioLegend) for 10 min at room temperature prior to antibody staining. For flow analysis of the tumor tissue, the tumor was isolated and digested into a single‐cell suspension by mechanical digestion followed by enzymatic digestion (1 mg/mL collagenase type IV (#C4‐28, Sigma‐Aldrich) with 0.2 mg/mL DNase I (#10104159001, Sigma‐Aldrich)) for 30 min at 37°C. The cells were filtered through a cell strainer, and RBC lysis was performed. Cells were stained with the antibodies below [clone] and analyzed by Cytek Aurora flow cytometer. PE anti‐mouse CD80 antibody [16‐10A1], APC anti‐mouse CD206 antibody [C068C2], FITC anti‐mouse F4/80 antibody [BM8], PB anti‐mouse CD45 antibody [30‐F11], PE/Cy7 anti‐mouse Ly‐6C antibody [HK1.4], PerCP anti‐mouse CD11c antibody [N418], PE anti‐mouse CD3 antibody [17A2], PE/Cyanine5 anti‐mouse CD4 antibody [RM4‐5], PE/Cy7 anti‐mouse CD8a antibody [S18018E], and BV605 anti‐mouse Ly‐6C antibody [HK1.4]. All antibodies were purchased from BioLegend.

### 4.9 Statistical Analysis

No data point was excluded. Normalization is indicated in figure captions to what the data was normalized. Data are presented with SD, SEM, or confidence intervals, which are indicated in figure captions. Sample size for each group and statistical methods used to assess significance (including details of one‐ or two‐way testing, repeated measures, type of pairwise comparisons, and P values) are provided in figures and figure captions. Origin Pro (v2024b) was used for statistical analyses of Figure 5c and Figure 6c. Biorender.com was used for all other statistical analyses.

## Author Contributions

S.S., M.K., and S.Y.E. conceived the study. S.S., M.C., E.C., Z.B., Seonghun Shin, and P.S.P. performed experiments. S.S. and J.L. interpreted and curated data. S.S. wrote the initial manuscript. S.S., M.C., M.K., E.C., J.L., Z.B., Seonghun Shin, P.S.P., and S.Y.E. revised the manuscript. S.Y.E. provided resources and supervised the study.

## Conflicts of Interest

The authors declare no conflicts of interest.

## Supporting information




**Supporting File**: adma74976‐sup‐0001‐SuppMat.docx.

## Data Availability

The data that supports the findings of this study are available in the Supporting Information of this article.
