# Supplementary material for: Photoacoustic Nanotagging for In Vivo Tracking and Optimization of Macrophage Immunotherapy in Solid Tumors
Source: Adv Mater. 2026 Sep 13;38(61):e74976. doi: 10.1002/adma.74976 (PMC13726695; doi:10.1002/adma.74976)
Supplement: Supplementary file 1 [file ADMA-38-e74976-s001.docx]

**Supporting Information**

**Photoacoustic Nanotagging for In Vivo Tracking and Optimization of Macrophage Immunotherapy in Solid Tumors**

**Seoyoon Song^1^, Melissa Cadena^1^, Myeongsoo Kim^1,2,3^, Euisuk Chung^3^, Jeungyoon Lee^3^, Zlata Brushevich^3^, Seonghun Shin^1^, Paul S. Pelkowski^1^, Stanislav Y. Emelianov^1,2,3,^***

^1^ Wallace H. Coulter Department of Biomedical Engineering, Georgia Institute of Technology and Emory University School of Medicine, Atlanta, Georgia 30332, United States

^2^ Petit Institute for Bioengineering and Biosciences, Georgia Institute of Technology, Atlanta, Georgia 30332, United States

^3^ School of Electrical and Computer Engineering, Georgia Institute of Technology, Atlanta, Georgia 30332, United States

* To whom correspondence should be addressed

**Figure S1.** Spectra of HBGNCs over a week period.

**Figure S2.** Spectra of HBGNC (before conjugation with IFNγ) and HBGNCγ (after conjugation).

**Figure S3.** Viability of HBGNC tagged (OD 1) macrophages over 48 hours. Data represent mean with SEM (n = 3). One-way ANOVA with Tukey’s multiple comparisons test. ns, not significant.

**Figure S4.** (a-d), Expression levels of MHCII (a), CD80 (b), CD86 (c), and CD206 (d) toward varying concentrations (0, 10, 50, 100 ng/mL) of free IFNγ and HBGNCγ. Data represent mean with SEM (n = 3). Unpaired two-tailed t-test.

**Figure S5.** Macrophage migration at 6 hour point.

(a) Representative confocal microscopy images of the migrated macrophages at 6-hour point. Scale bars: 100 μm. (b) Number of migrated macrophages (normalized) across different treatment groups at 6-hour point. Data represent mean with SEM (n = 3, each data point represents the average number of macrophages from three different random points). One-way ANOVA with Tukey’s multiple comparisons test.

**Figure S6.** Concentrations of IL-6 (a), nitrite (b), and TNF-α (c) in culture supernatant at 8 h post-LPS treatment. Data represent mean with SEM (n = 3). Two-way ANOVA with Tukey’s multiple comparisons test.

**Figure S7.** Concentrations of IL-6 (a), nitrite (b), and TNF-α (c) in culture supernatant at 24 h post-LPS and MPLA treatments. Data are normalized to control (PBS treatment) of PBS-Mac group. Data represent mean with SEM (n = 4).

**Figure S8.** (a, b) PA signal over two days post tagging macrophages with HBGNCs (OD 1, 1000 cells/μL). US/PA overlay images (a) and PA signal quantification graph (b). Data represent mean with SEM (n = 3). One-way ANOVA with Tukey’s multiple comparisons test. ns, not significant. Scale bar: 1 mm.

**Figure S9.** Viability of macrophages after 1000 laser pulses. Data represent mean with SEM (n = 2-3). One-way ANOVA with Tukey’s multiple comparisons test.

**Figure S10.** US/PA monitoring of locally injected macrophages.

(a) Treatment and imaging regime. Subcutaneous CT26 tumor was inoculated on Day 0. US/PA imaging was performed every other day from Day 5 post inoculation for a total of 5 times. Macrophages were intratumorally injected on Day 6. (b) US/PA overlay images of the tumors with different treatments. Dotted lines indicate the tumor periphery. Scale bar: 2 mm. (c), Quantified PA signals from (b). Data represent mean with SEM. (d) Tumor volume monitoring. Data represent mean with SEM. (e), Body weight changes. Data represent mean with SEM. n = 3 mice for PBS and n = 4 mice for other groups for (b-e).

**Figure S11.** Body weight changes over the course of experiment (iv injection of macrophages). Data represent mean with SEM. n = 4 mice per group.

**Figure S12.** Tumor volume monitoring of mice received local injections of PBS or LPS as outlined in Figure 6a. Data represent mean with SEM. n = 8 mice per group.

**Figure S13.** PA signal persistence comparison between HBGNC-Mac and HBGNCγ-Mac from combination therapy regime in Figure 6. PA signals were normalized to D+7 signal. Data represent mean with SEM. n = 8 mice per group.

**Figure S14.** (a) Body weight changes and (b) body conditioning scores of mice in each treatment group over the course of experiment (iv injection of macrophages + LPS). Data represent mean with SEM. n = 4 mice per group.

**Figure S15.** Evaluation of anti-metastatic effects.

(a) Representative photographs of the lung from fresh control group mice and HBGNCγ-Mac group mice with tumor remissions after intravenous injection of CT26 cells. (b) Number of metastatic nodules (surface-exposed) from the lungs of fresh control and HBGNCγ-Mac groups after intravenous injection of CT26 cells. Data represent mean with SEM. (c) Representative lung bright field scans (H&E) of fresh control and HBGNCγ-Mac groups after intravenous injection of CT26 cells. n = 2 rechallenged mice per group.

**Figure S16.** Tumor microenvironment analysis on Day 7.

(a) Number of adoptively transferred macrophages per mg tumor. (b) % of CD80^+^ activated macrophages among (a). (c) Number of total CD45^+^ leukocytes per mg tumor. (d) Number of total CD11c^+^ dendritic cells per mg tumor. (e) Number of total Ly-6C^+^ monocytes per mg tumor. (f) % of CD80^+^ activated monocytes among (e). (g) Number of total F4/80^+^ macrophage per mg tumor. h, % of CD80^+^ activated macrophages among (g). (i) Number of total CD3^+^ T cells per mg tumor. (j) % of CD4^+^ T cells among (i). (k) % of CD8^+^ T cells among (**i**). Data represent mean with SEM. n = 2-4 mice per group. One-way ANOVA with Tukey’s multiple comparisons test.
